# Double Contrast-Enhanced Ultrasonography in Preoperative T Staging of Gastric Cancer: A Comparison With Endoscopic Ultrasonography

**DOI:** 10.3389/fonc.2019.00066

**Published:** 2019-02-12

**Authors:** Liang Wang, Zhe Liu, Hongju Kou, Huiliao He, Bo Zheng, Lingling Zhou, Yan Yang

**Affiliations:** ^1^Department of Ultrasound, The Second Affiliated Hospital and Yuying Children's Hospital of Wenzhou Medical University, Wenzhou, China; ^2^Wenzhou Institute of Biomaterials and Engineering, Chinese Academy of Sciences, Wenzhou, China; ^3^Department of Gastroenterology, The Second Affiliated Hospital and Yuying Children's Hospital of Wenzhou Medical University, Wenzhou, China; ^4^Department of Pathology, The Second Affiliated Hospital and Yuying Children's Hospital of Wenzhou Medical University, Wenzhou, China

**Keywords:** ultrasonography, endoscopic ultrasonography, gastric cancer, surgery, histopathology

## Abstract

**Objective:** To compare the precision of double contrast-enhanced ultrasonography (DCEUS) to endoscopic ultrasonography (EUS) in preoperative T staging of gastric cancers.

**Methods:** This retrospective study consisted of 158 pathologically confirmed gastric cancer patients. All patients underwent DCEUS (intravenous contrast-enhanced ultrasonography combined with oral contrast-enhanced ultrasonography) and endoscopic ultrasonography (EUS) preoperatively. The histopathological findings of resected specimens were compared with the results of DCEUS and EUS retrospectively.

**Results:** The accuracy of DCEUS and EUS in evaluating the T staging of gastric cancer were 82.3% (T1 62.5%,T2 84.4%,T3 87.9%,T4 91.3%) and 76.6% (T1 84.4%,T2 82.2%,T3 72.4%,T4 65.2%), respectively. There were no significant differences between the methods for the overall T staging accuracy (χ^2^ = 1.569, *P* = 0.210). But EUS was superior to DCEUS for T1 stage (χ^2^ = 3.925, *P* = 0.048) and DCEUS was superior to EUS for T3 stage (χ^2^ = 4.393, *P* = 0.036) and T4 stage (χ^2^ = 4.600, *P* = 0.032).

**Conclusion:** DCEUS is a convenient and noninvasive method with high precision, which can be used as the primary imaging technique for advanced gastric cancer T staging. In early gastric cancer, we should prefer EUS. Two methods are complementary for assessing tumor invasion depth of gastric cancer.

## Introduction

Gastric carcinoma is a highly lethal malignant tumor. It is a serious public health problem in Eastern Asia, Eastern Europe, Central and South America (1). Gastric cancer ranks the fourth among all cancers (nearly 1,000,000 new cases per year) and ranks the third among all cancer deaths worldwide (2, 3). Despite recent improvement in diagnosis and therapeutic methods, prognosis of gastric cancer remains poor (4). Surgery is still the right choice for the malignancy (5). Accurate preoperative staging to select a reasonable range of surgical and adjuvant therapy program, to avoid over-treatment or inadequate treatment are essential (6, 7). The depth of tumor invasion is an important indicator for predicting a patient's prognosis when suffering from gastric carcinoma (8). So, it is important to explore reliable and effective techniques for preoperative T staging of gastric cancer.

Many modalities, such as barium radiography, gastroendoscopy, CT, and MRI are used to stage gastric tumors (9, 10). Nevertheless, until now, no suitable tumor screening method for gastric carcinoma has been suggested by the World Health Organization (11). EUS is regularly utilized to identify and stage gastrointestinal cancers and provides detailed images (12, 13). Many researchers have studied the function of EUS in the preoperative staging of gastric cancer, and EUS is regularly contemplated as the primary imaging tool for regional staging of gastric carcinoma as compared with other methods (13–16). However, patient discomfort and risk of cross-infection hamper its application.

We need to identify a noninvasive, simple, economic and reliable approach in modern times. Double contrast-enhanced ultrasonography (DCEUS) has developed as an innovative modality to screen the diseases of gastrointestinal tract such as gastric tumors and rectal neoplasms in China (17, 18). SonoVue is an intravenous contrast agent of sulfur hexafluoride microbubbles (19). Combining ultrasonic oral contrast agent (UOCA) and SonoVue in patient examination, it is easy for us to detect gastric carcinoma, giving a precise T-staging. This study reviewed 158 gastric carcinoma cases and compared DCEUS with EUS in surgical outcomes to investigate the importance of DCEUS in the preoperative T-staging of the disease.

## Methods

This study was carried out in agreement with the Declaration of Helsinki. The protocol was sanctioned by the Research Ethics Committee of the Second Affiliated Hospital of Wenzhou Medical University. All patients gave written informed consent.

### Patients

Between January 2015 and July 2017, 183 consecutive subjects were diagnosed with gastric cancer at our Hospital. The inclusion criteria for this study were: ①gastric carcinoma as confirmed by endoscopic biopsy; ②without previous chemotherapy, radiotherapy, immunotherapy or treatment; ③patients were examined by both DCEUS and EUS a week before surgical resections. The exclusion criteria included: ①unresectable tumors with widespread metastasis (16 cases); ②elderly patients with contraindications for surgery (9 cases). A total of 158 patients were included in the final study [52 females, 106 males, average of 59.5 ± 10.6 years of age (range 33–80)].

#### Equipment's and Contrast Agents

DCEUS examinations were performed with Acuson Seioquoia 512 ultrasound system, equipped with contrast pulse sequencing (CPS) technology; UOCA Xinzhang®(Huqingyutang, HangZhou, China) was made from a soya derivative; Intravenous contrast agent SonoVue (Bracco, Milan, Italy)–a suspension of sulfur hexafluoride microbubbles.

EUS studies were performed with EndoEcho system (Olympus, Japan): Model for the host EU-M2000; endoscopic ultrasonography for the Olympus GF-UM 2000-ring endoscopic ultrasound scan, the department tip diameter of 12.7 mm, pipe pliers diameter of 2.2 mm, scan range of 360°; Olympus UM-DP12-25R, and UM-DP20-25R ultrasonic micro-probe; ultrasonic probe drive MAJ-935; MH-303 bladders (Japan); sterile degassed water (our hospital).

### Examination and Observation

#### DCEUS Examination

All patients were fasting more than 8 h and received atropine sulfate (0.05 mg/kg) intramuscularly 30 min before examinations to minimize the gastric peristalsis. Firstly, a basic 2D ultrasound examination was performed by using 4V1 probe to identify each gastric lesion. Then the patients ingested UOCA (500 ml) which fills the stomach, and were examined in the supine, and both decubitus positions. The tumors were observed, the sizes of masses were determined, the echoic features and shape of lesions were described. Further steps were performed following a bolus 2.4 ml Sonovue injection. The contrast pulse sequencing (CPS) mode was used when we performed DCEUS. The settings were as follows: acoustic power, −15 to −21 dB; transmit frequency, 1.5 MHz; frame rate, 17–20. A low (<0.2) mechanical index was used to prevent microbubble disruption. The enhancement patterns of the arterial phase, the venous phase, and the late phase were stored. The storage is up to 5 min. All baseline 2D and dynamic DCEUS images were recorded on tapes. The images were then assessed by two impartial off-site sonographers. Both of them were blinded to the clinical data, pathology results and other imaging findings of the patients at the time of the analysis.

#### EUS Examination

Patients were fasting for more than 8 h. The Olympus GF-UM2000 EUS scan-ring were inserted into the level of the duodenum, detailed inspection from the beginning of the duodenum to the esophagus, including duodenum, pylorus, antrum, gastric body, fundus, cardia, esophagus, and the organs around digestive tract, such as the pancreas, part of the liver, spleen, retroperitoneum around the aorta, mediastinum were all observed. The endoscope was advanced beyond the tumor mass. Serial images were obtained when the transducer was pulled back. In order to increase the surface contact and improve the acoustic window, the transducer was surrounded by an inflatable balloon which was filled with deaerated water (20). The sizes of lesions, borders, depths, surrounding organs were all observed. Endoscopic images of the target lesions were subsequently analyzed by two other independent off-site physicians having over 10 years experience. Both of them were unaware of the patients' clinical symptoms, signs, laboratory tests, other imaging results.

**Figure 1:**
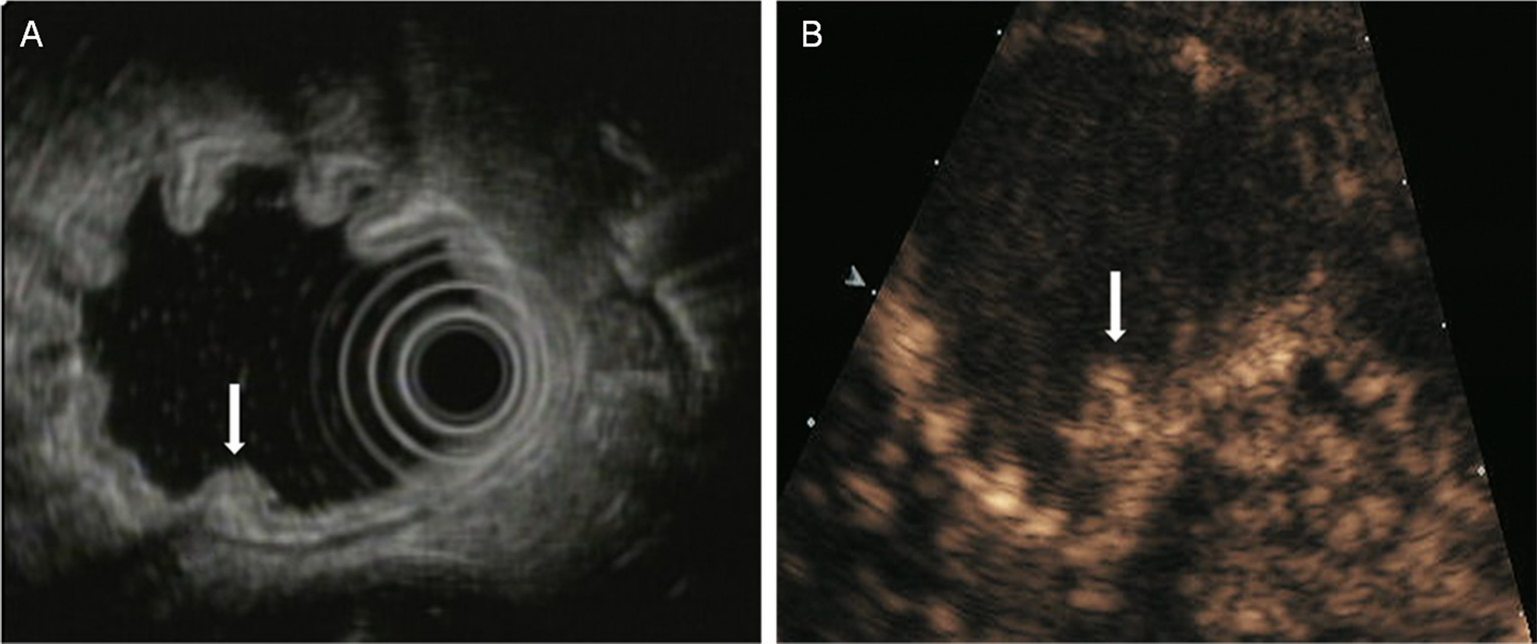
A case with T1 tumor located in the posterior wall of antrum. **(A)** EUS showed an small elevated lesion (arrow) involving the antral mucosa and submucosa. **(B)** DCEUS showed the lesion (arrow) invaded into the submucosal layer.

**Figure 2:**
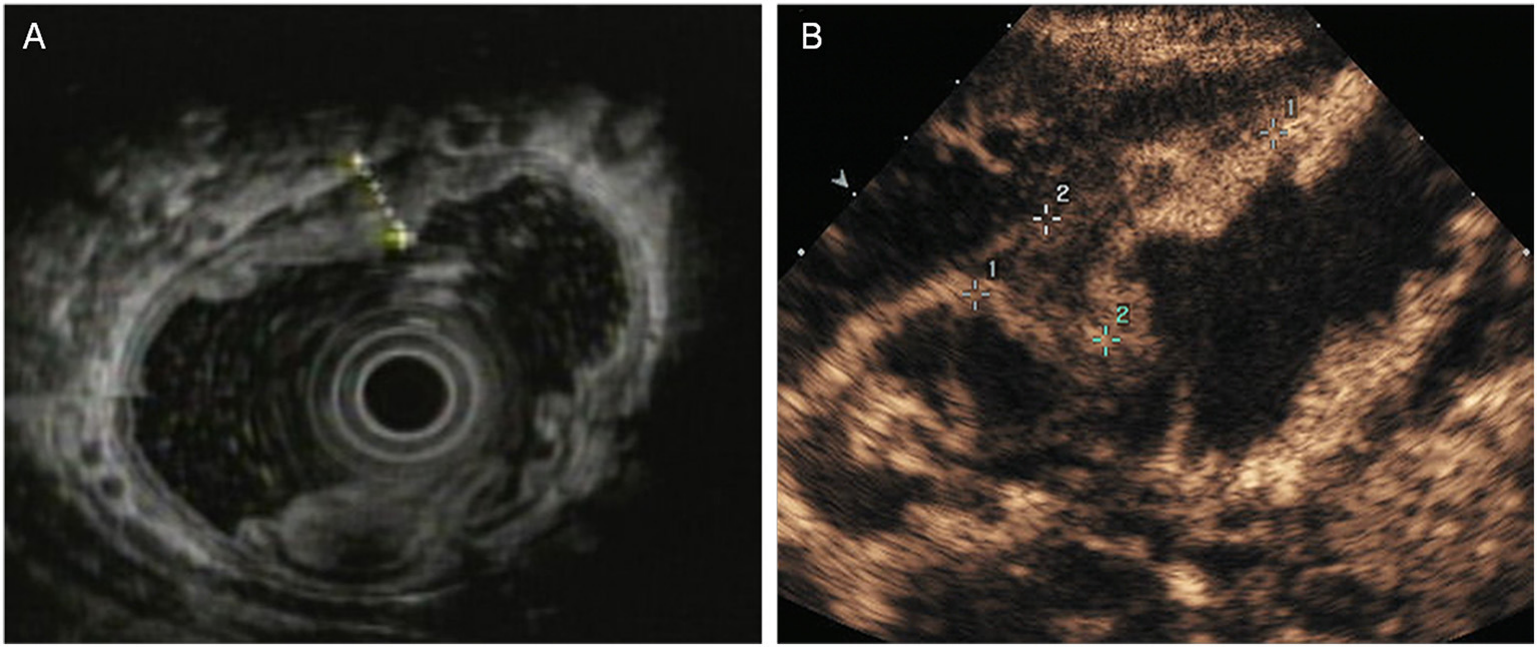
A case with T2 tumor located in the anterior wall of the gastric body. **(A)** EUS showed an ulcerative lesion involving the muscularis propria (fourth layer). The serosal layer appears intact. **(B)** DCEUS image showed the lesion invaded into the muscularis propria. The lesion didn't penetrate the serosa.

**Figure 3:**
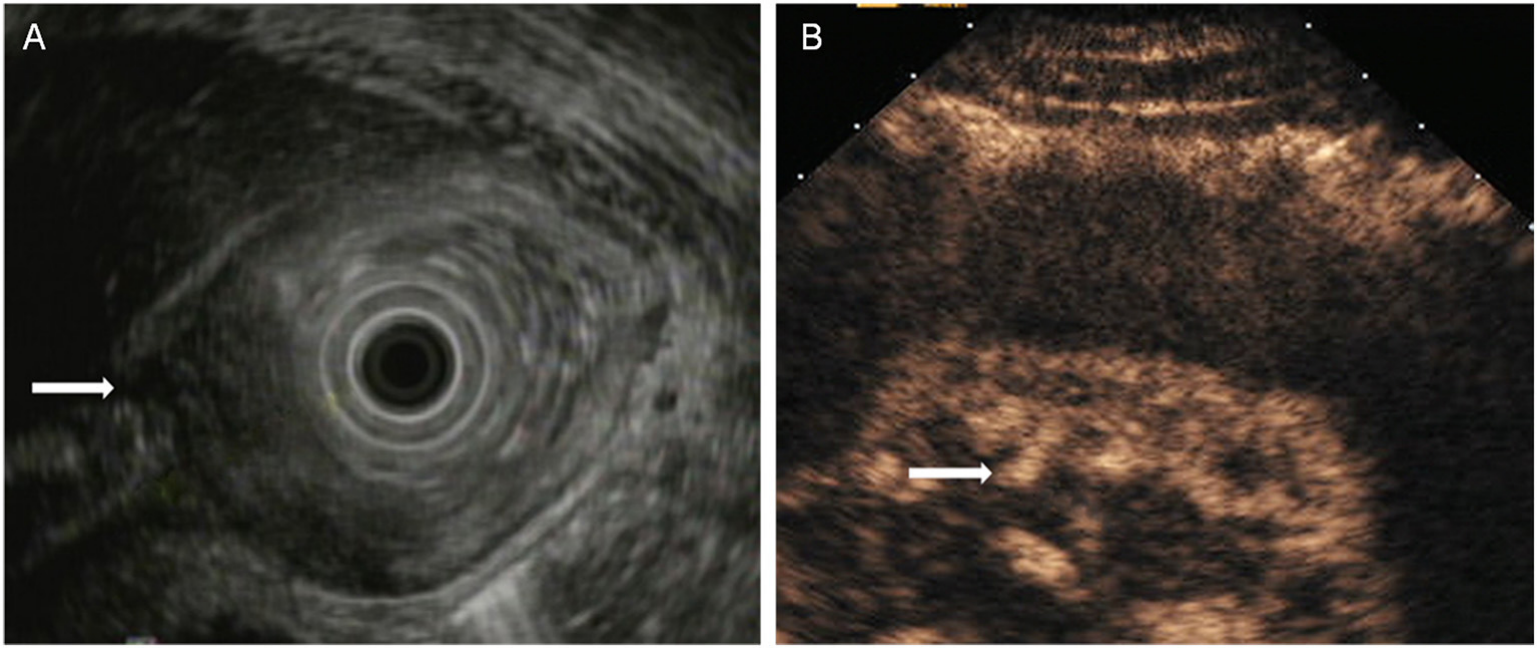
A case with T3 tumor located in the gastric angle. **(A)** EUS showed an large lesion with a thickness of approximately 3 cm penetrated the serosal layer. **(B)** DCEUS showed the lesion penetrated the serosa (arrow) with disappearance of all layers.

**Figure 4:**
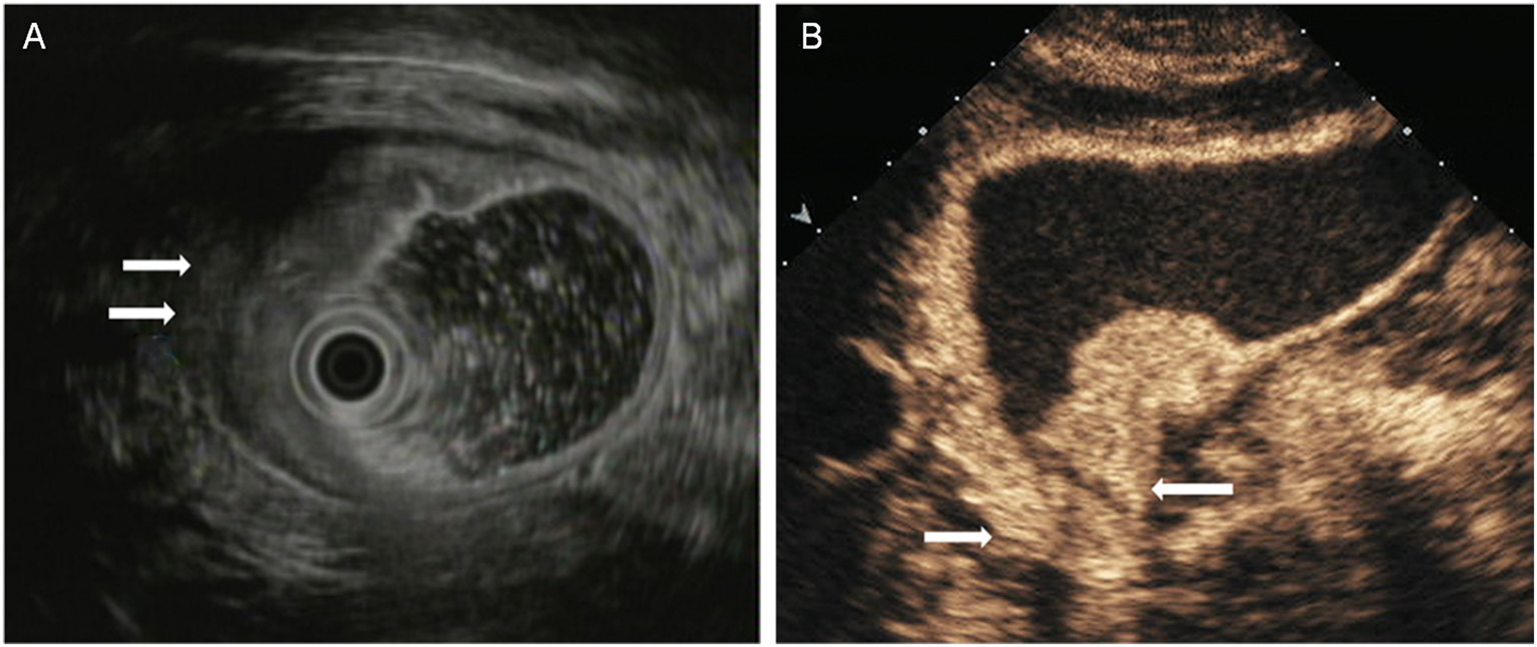
A case with T4 tumor located in the antrum. **(A)** EUS showed an lesion penetrated the serosal layer and invaded into adjacent tissues (arrow). **(B)** DCEUS showed the lesion infiltrated to duodenum bulb (arrow).

The T staging criteria of both DCEUS and EUS are based on the five-story structure of the gastric wall (21, 22). T1: tumor-infiltrating limited to the first 1 to 3 layers, that is located in mucosa or submucosa (Figure 1); T2: tumor invasion to the fourth layer, that is up to the inherent muscle (Figure 2); T3: tumor invasion to the fifth layer, that is, invasion to serosa layer (Figure 3); T4: tumor invasion to the serosa adjacent tissues or organs (Figure 4).

The surgical specimens were transported to the pathology department after operations. The microscopic staging of the resected specimens were performed by the pathologist (L Z, with 8 years of proficiency), who was unaware of the DCEUS and EUS findings.

### Statistical Analysis

Data analysis was performed by using SPSS version 22.0 software. The diagnostic performance change from DCEUS to EUS was measured by chi-square test. All *P*-values were derived from 2-tailed tests, and *P* < 0.05 was selected to designate a statistically significant difference.

## Results

All 158 patients undertook surgery. The diameters of resected gastric lesions were in the range of 1.2–11.6 cm (mean 5.2 ± 1.6 cm). Among 158 patients, the tumors were mostly located in the antrum and pylorus region (*n* = 66), followed by the proximal cardia region (*n* = 50).

The histopathological classifications were as follows: well-differentiated adenocarcinoma = 22 cases, moderately differentiated adenocarcinoma = 33 cases, poorly differentiated adenocarcinoma = 65 cases, undifferentiated adenocarcinoma = 13 cases, signet-ring cell carcinoma = 16 cases, mucinous adenocarcinoma = 8 cases, and squamous carcinoma = 1 case. There were 32 and 126 cases of early and advanced gastric cancer, respectively.

Tumor depth invasion was categorized as follows: T1 = 32 cases, T2 = 45 cases, T3 = 58 cases, and T4 tumors = 23 cases. One hundred and thirty cases were correctly staged by DCEUS and 121 cases were correctly staged by EUS, respectively. For DCEUS, the overall T staging accuracy was 82.3%, with each stage as follows:T1 staging accuracy = 62.5%, T2 staging accuracy = 84.4%, T3 staging accuracy = 87.9%, and T4 staging accuracy = 91.3%. A total of 19 cases were overstaged (12 patients with T1 overstaged as T2; 4 patients with T2 overstaged as T3; 3 patients with T3 overstaged as T4) and 9 cases were understaged (3 patients with T2 understaged as T1; 4 patients with T3 understaged as T2; 2 patients with T4 understaged as T3) (Table 1). For EUS, the overall T staging accuracy was 76.6%, with each stage as follows: T1 = 84.4%, T2 = 82.2%, T3 = 72.4%, and T4 = 65.2%. A total of 17 cases were overstaged (5 patients with T1 overstaged as T2; 6 patients with T2 overstaged as T3; 6 patients with T3 overstaged as T4) and 20 cases were understaged (2 patients with T2 understaged as T1; 10 patients with T3 understaged as T2; 8 patients with T4 understaged as T3) (Table 2).

**Table 1:** The results of T staging by DCEUS compared with postoperative pathological findings.

	**DCEUS**					
**Pathology**	**T_**1**_**	**T_**2**_**	**T_**3**_**	**T_**4**_**	**Total**	**Accuracy (%)**
T_1_	20	12			32	62.5
T_2_	3	38	4		45	84.4
T_3_		4	51	3	58	87.9
T_4_			2	21	23	91.3

*DCEUS, double contrast-enhanced ultrasonography. The overall accuracy of DCEUS in T staging was 82.3% (130 of 158 patients)*.

**Table 2:** The results of T staging by EUS compared with postoperative pathological findings.

	**EUS**					
**Pathology**	**T_**1**_**	**T_**2**_**	**T_**3**_**	**T_**4**_**	**Total**	**Accuracy (%)**
T_1_	27	5			32	84.4
T_2_	2	37	6		45	82.2
T_3_		10	42	6	58	72.4
T_4_			8	15	23	65.2

*EUS, endoscopic ultrasonography. The overall accuracy of EUS in T staging was 76.6% (121 of 158 patients)*.

Statistically, there was no significant difference between two methods for the overall T staging accuracy (χ^2^ = 1.569, *P* = 0.210). But EUS was superior to DCEUS for T1 stage (χ^2^ = 3.925, *P* = 0.048); DCEUS was superior to EUS for T3 stage (χ^2^ = 4.393, *P* = 0.036) and T4 stage (χ^2^ = 4.600, *P* = 0.032) (Table 3).

**Table 3:** Comparison of the two methods in T staging of gastric cancer.

**T stage**	**DCEUS (%)**	**EUS (%)**	**χ^2^**	***P***
T_sum_	82.3	76.6	1.569	0.210
T_1_	62.5	84.4	3.925	0.048^*^
T_2_	84.4	82.2	0.080	0.777
T_3_	87.9	72.4	4.393	0.036^*^
T_4_	91.3	65.2	4.600	0.032^*^

* *P < 0.05*.

## Discussion

Gastric cancer is a common malignancy of the digestive tract. Its treatment and survival rates are associated with the early diagnosis and accurate clinical staging for a reasonable range of surgery.

Surgical resection of gastric cancer remains the only available treatment and depends on the stage of the disease at presentation. Some early gastric cancer (T1) may be treated with endoscopic submucosal dissection (ESD) and endoscopic mucosal resection (EMR). Lymph node dissection is required for advanced gastric cancer (T2 stage and above). For primary tumors or metastases that directly invade adjacent organs, the affected organs should be removed jointly. And for advanced gastric cancer, adjuvant and neoadjuvant chemotherapy, either alone or in combination with radiation therapy, have been shown to improve survival rates (23). Therefore, accurate preoperative T stage is very important for planning the optimal surgical procedure.

Reliable preoperative T staging methods that are consistent with the pathological specimens are necessary prior to developing a treatment plan. However, each of the currently used methods have limitations and no single staging method is acknowledged as the method of choice. Consequently, National Comprehensive Cancer Network practice guidelines for gastric carcinoma do not suggest explicit methods and recommend the use of various modalities supplementary as staging technique (24).

EUS combines the advantages of endoscopy and ultrasound. It can not only display the location, shape, size, internal echo of the tumor, but also provides detailed images of the malignancy infiltrating depth. For the T staging of gastric carcinoma, EUS has been identified as the preferred diagnostic method (25, 26). There are however, several opposing statements about the reliability of EUS in determining the T stage since described values for EUS diagnostic accuracy in overall T staging ranged from 42.6 to 87.7% (26–28). In this research, the general accuracy of EUS was 76.6%; Among the different stages, the accuracy was 84.4% for T1, 82.2% for T2, 72.4%for T3, and 65.2% for T4. A total of 17 (10.8%) cases were overstaged and 20 (12.7%) cases were understaged.

Traditional transabdominal ultrasound is unable to adequately stage the depth of gastric tumors, due to variations of wall thickening frequently being too delicate to visualize (29). Double contrast-enhanced ultrasonography is a relatively new method. It is a transabdominal ultrasound method utilizing both intraluminal and intravenous contrast to improve ultrasonographic visualization. The use of UOCA can distend the gastric lumen and displace the air in the stomach, therefore helping to display mucosal lesions (30). The use of intravenous contrast can demonstrate blood perfusion of the tumors and enhance visualization of lesions through the arterial phase to identify the invasion depth. Thus, DCEUS can display morphologic appearances and perfusion characteristics of both normal and abnormal structures (31). UOCA can discharge intragastric air and form a homogeneous distribution of ultrasonic transmission surface. This may lead to a reduction in ultrasonic artifacts and provides a good acoustic window, to increase the detection rate of the gastric lesions. But it is difficult for oral contrast enhancd ultrasound to differentiate tumor tissues from inflammation and fibrosis due to the small acoustic impedance difference and the limitation of resolution (32). And this is the most common reason for overestimation or underestimation while using single oral contrast enhancd ultrasound (20, 33, 34). Angiogenesis and infiltration are essential for the invasive growth of tumors (35). Single oral contrast enhancd ultrasound cannot show the microvascular perfusion of the tumors. As a blood pool agent, SonoVue can, through blood circulation, enter the capillary of gastric lesions. It can create strong echoes over a range of frequencies routinely utilized in medical ultrasound examinations (19). Combining UOCA and SonoVue is useful to stage gastric malignancy before surgery. In this study, the general accuracy of DCEUS for T staging was 82.3%; Among the different stages, the accuracy was 62.5% for T1, 84.4% for T2, 87.9% for T3, and 91.3% for T4. Tumors enhanced in the arterial phase and agents washed out in the venous phase, which made the borders of lesions clearer. Therefore, the tumor's contour and invasive depth can be easily identified. Overestimation and underestimation also existed in T staging using DCEUS. A total of 19 (12.0%) cases were overstaged and 9 (5.7%) cases were understaged.

**Figure 5:**
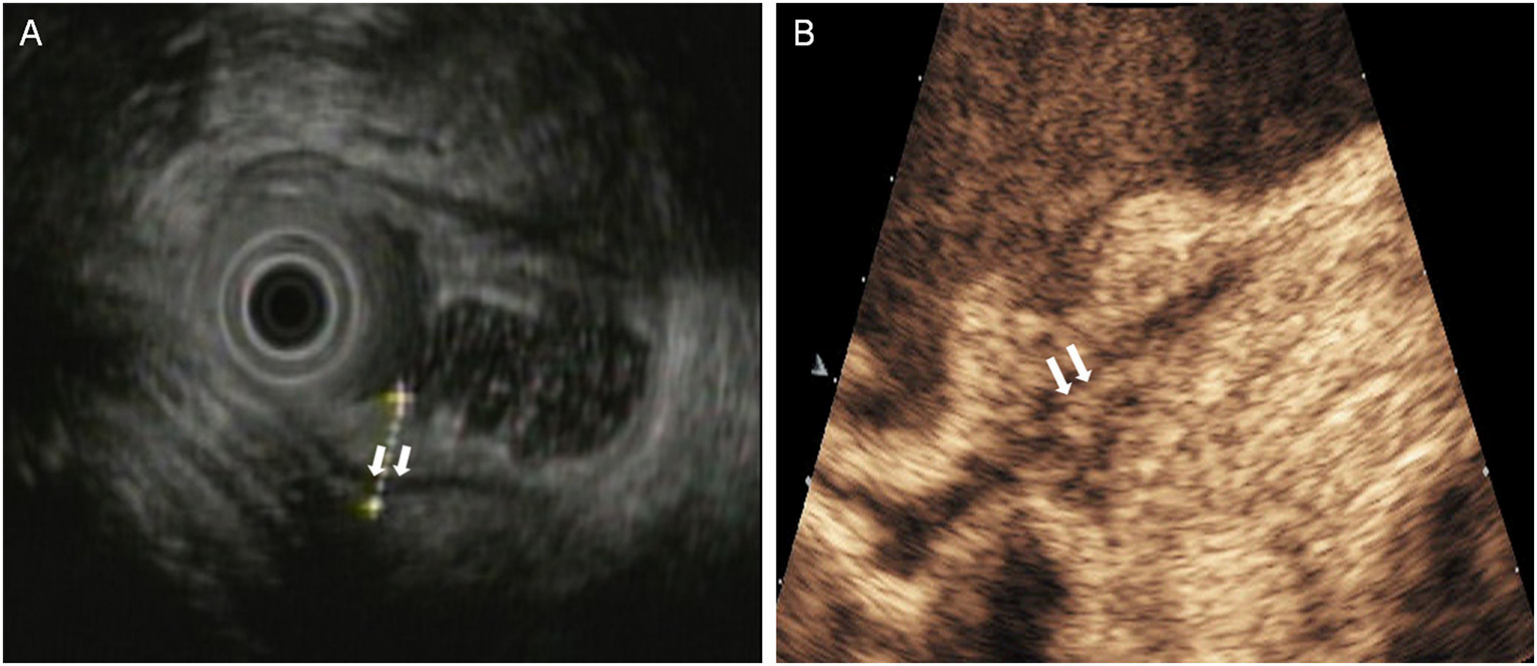
A case with T1 tumor was overstaged by DCEUS but was accurately staged by EUS. **(A)** EUS showed a lesion invaded into the submucosa, and the muscularis propria was intact (arrow), but **(B)** DCEUS showed the lesion invaded into the muscularis propria (arrow) in error and overstaged it as T2 tumor.

**Figure 6:**
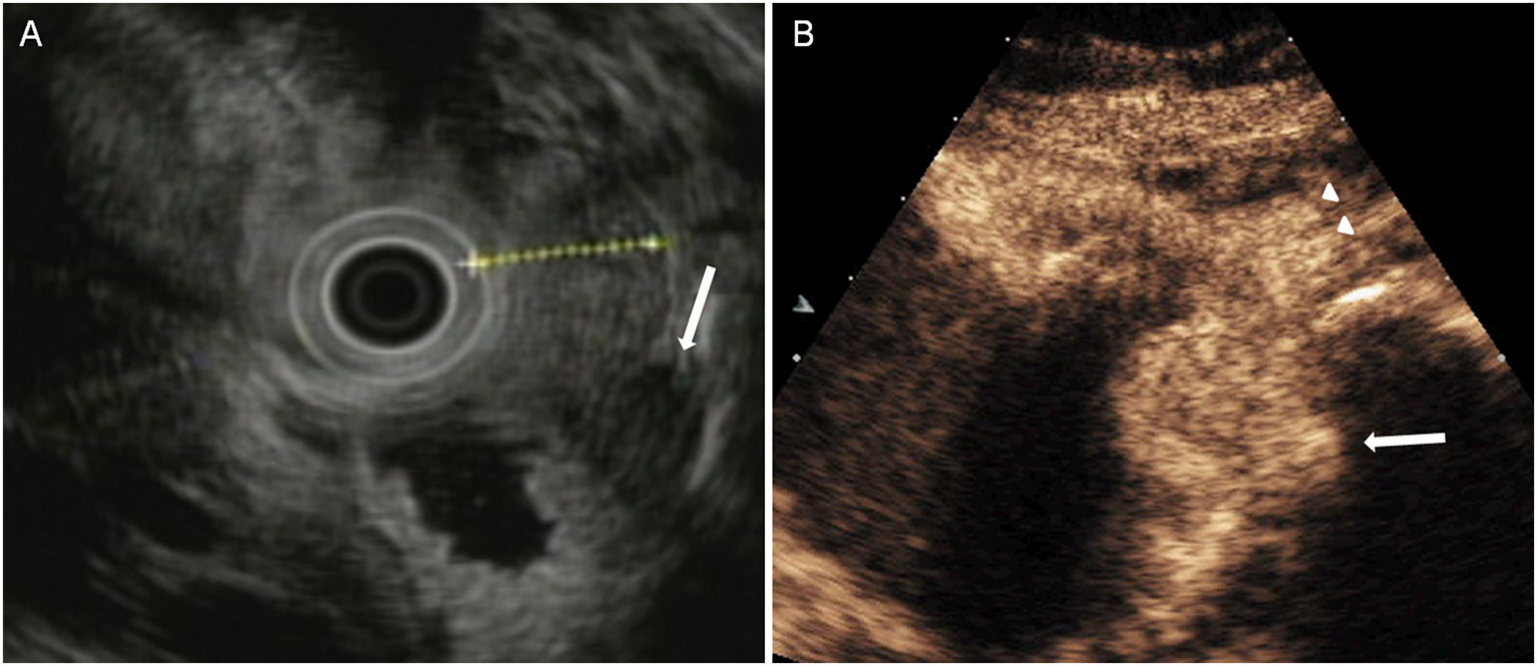
A case with T4 tumor was understaged by EUS but was accurately staged by DCEUS. **(A)** EUS showed an lesion penetrated the serosa (arrow) and understaged it as T3 tumor. **(B)** DCEUS showed the lesion not only penetrated the serosal layer (arrow) but also infiltrated to adjacent tissues (triangle).

In this study, the overall accuracy of DCEUS was similar to that of EUS in determining the T stage. But EUS was superior to DCEUS for T1 stage and DCEUS was superior to EUS for T3 and T4 stage. EUS is used to visualize the lesion surface, portraying interference of abdominal fat and gas in the stomach. Thus, EUS is more accurate in identifying stages early in well-differentiated carcinomas (Figure 5) (36). The accuracy of EUS in late stage tumors was reduced. A lesion-by-lesion analysis revealed that overestimation of EUS mostly present in the cases of malignancy with ulcerative type. Using EUS it is difficult to differentiate fibrosis and inflammation from tumors, which is a common cause of misreading of depth in ulcerative malignancy. Moreover, we found underestimation of invasion depth mostly present in the cases of tumors with diameters >5 cm. This maybe due to the tumors being too large and the frequency of EUS being relatively high, it was hard for EUS to display the whole lesions or to show the views of the maximum depths of infiltration (Figure 6). Contrariwise, DCEUS is better for determining the stage of advanced gastric cancer due to the rich blood supply. The advantage of DCEUS is its high contrast resolution, which can be used to distinguish tumors from normal tissue. Hence, it is sensitive to lesion detection, characterization, and staging. What's more, it can show the relationship of the lesion's vasculature and the gastric wall, in addition to their contours. The lack of vascularity in early gastric cancer leads to its low accuracy. In addition, other factors may also influence its accuracy, such as unsatisfied filling of gastric cavity, artifacts caused by gastric peristalsis.

In our opinion, DCEUS is a viable supplement to the preoperative work-up of biopsy-proven gastric cancer in the preoperative staging of the disease. The findings of DCEUS can be valuable and additive for the suitable treatment plans for gastric cancer patients, in particular for the elder population which invasive EUS carries risks. Furthermore, the cost of doing both EUS and DCEUS is not considerable in our hospital, and the additional information makes the cost of performing both tests worthwhile.

This study was a retrospective study, only enrolling patients referred to our hospital for surgery. This represent a bias issue that may affect accurate evaluation. A prospective study should be performed to avoid the bias in future research.

In conclusion, as a convenient and noninvasive technique with high accuracy, DCEUS can be used as the primary imaging modality for the T staging of advanced gastric cancer. In early gastric cancer, we should prefer to EUS. Two methods are complementary for assessing tumor invasion depth of gastric cancer.

## Author Contributions

LW and YY: designed this study. LW, HK, and BZ: acquired the data. ZL, HH, and LZ: interpreted the data. LW: wrote and edited the manuscript. All authors reviewed the manuscript.

### Conflict of Interest Statement

The authors declare that the research was conducted in the absence of any commercial or financial relationships that could be construed as a potential conflict of interest.
